# The Rutgers Omnibus Study: Protocol for Quarterly Web-Based Surveys to Promote Rapid Tobacco Research

**DOI:** 10.2196/58203

**Published:** 2024-10-16

**Authors:** Michelle T Bover Manderski, William J Young, Ollie Ganz, Cristine D Delnevo

**Affiliations:** 1 Institute for Nicotine and Tobacco Studies Rutgers Biomedical and Health Sciences (Rutgers Health) Rutgers, The State University of New Jersey New Brunswick, NJ United States; 2 Department of Biostatistics and Epidemiology School of Public Health Rutgers, The State University of New Jersey Piscataway, NJ United States; 3 Department of Health Behavior, Society, and Policy School of Public Health Rutgers, The State University of New Jersey Piscataway, NJ United States

**Keywords:** survey, tobacco, nicotine, young adults, adults, protocol, Rutgers Omnibus Study, Amazon Mechanical Turk, MTurk

## Abstract

**Background:**

Rapid and flexible data collection efforts are necessary for effective monitoring and research on tobacco and nicotine product use in a constantly evolving marketplace. The Rutgers Omnibus Survey (1) provides timely data on awareness and use of new and emerging tobacco products among adults in a rapid manner, (2) provides a platform for measurement experiments to help develop and refine measures of tobacco use that reflect the current marketplace, and (3) generates pilot data for grant applications and scientific manuscripts.

**Objective:**

This study aims to document the first 2 years of the Rutgers Omnibus Study through the reporting of methodology, fielding summaries, and sample characteristics.

**Methods:**

Launched in February 2022 and fielded quarterly thereafter, we survey convenience samples of 2000 to 3000 US adults aged 18-45 years recruited from Amazon Mechanical Turk (MTurk) using the MTurk Toolkit by CloudResearch. The questionnaire includes core and rotating modules and is designed to take approximately 10 minutes to complete through Qualtrics. The fielding duration is approximately 10 days per wave. Each wave includes both unique and repeating participants, and responses can be linked across waves by an anonymous ID.

**Results:**

Sample sizes ranged from 2082 (wave 8, December 2023) to 2989 (wave 1, February 2022), and the 8-wave longitudinal dataset included 10,334 participants, of whom 2477 had 3 or more data points. The cost per complete at each wave was low, ranging from US $2.46 to US $3.27 across waves. Key demographics were consistent across waves and similar to that of the general population, while tobacco product trial and past–30-day use were generally higher.

**Conclusions:**

The Rutgers Omnibus Study is a quarterly survey that is effective for rapidly assessing the use of emerging tobacco and nicotine products and can also be leveraged to conduct survey experiments, generate pilot data, and address both cross-sectional and longitudinal research questions.

**International Registered Report Identifier (IRRID):**

RR1-10.2196/58203

## Introduction

Tobacco companies in the United States have undergone dramatic changes in the marketing and manufacturing of tobacco. Whereas tobacco control efforts have historically focused on cigarette smoking, the marketplace has evolved to include numerous products such as electronic nicotine vaping devices and “tobacco-free” nicotine pouches. Today, the tobacco marketplace is in constant flux, with the emergence of new products, flavors, and packaging styles on a regular basis [1,2], and tobacco use patterns are changing in turn [3,4].

Traditional health behavior surveys, such as the Behavioral Risk Factor Surveillance System (BRFSS), have been slow to respond to the changing marketplace. The most recent BRFSS survey (2022) did not include questions about the newest products, and the limited data about tobacco that are collected are typically not available for many months after data collection. Tobacco-centric surveys, such as the Population Assessment of Tobacco or Health Study (PATH), the National Youth Tobacco Survey (NYTS), and the Tobacco Use Supplement to the Current Population Survey (TUS), have improved our ability to monitor perceptions and use of a wider variety of products, but each wave of data takes months to collect and even longer to release for public analysis. While these studies are ideal for generating nationally representative, generalizable estimates of tobacco use, they are not well suited for rapid and flexible data collection to capture changes in tobacco products, tobacco marketing, and tobacco use in the United States.

With the growing variety of tobacco and nicotine products, there is a critical need to develop and test valid and precise measures for assessing use patterns and perceptions. For example, an investigation into measures of modern oral nicotine product use found substantial variation in the phrasing of questions about these products, calling for the development of common and validated measures [5]. However, large national survey systems are not suited for this purpose. Instead, smaller studies that use convenience samples are sufficient for conducting survey methods experiments [6]. Similarly, academic researchers can also use convenience samples to generate pilot data in support of a funding proposal. However, developing and fielding a separate study for each measurement question or pilot inquiry is both time and cost prohibitive.

In response to these methodological challenges, we designed the Rutgers Omnibus Survey, a serial web-based survey of adults aged 18-45 years designed to capture awareness and use of new and emerging tobacco products. The Rutgers Omnibus Survey serves 3 major functions. First, it provides timely data on the awareness and use of new and emerging tobacco products among adults in a rapid manner. Second, it provides a platform for measurement experiments to help develop and refine measures of tobacco use that reflect the current marketplace. Finally, it generates data to serve as pilot data for grant applications and timely scientific manuscripts among investigators at the Rutgers Institute for Nicotine and Tobacco Studies. The aim of this paper is to document the first 8 waves of the Rutgers Omnibus Survey protocol and describe the characteristics of the study samples.

## Methods

### Overview

The Rutgers Omnibus is fielded quarterly; although there are minor differences across waves, the primary aspects of survey development and administration are consistent. Each wave’s survey is programmed in Qualtrics, a secure web-based survey program, and extensively tested by research staff. All waves recruit a convenience sample of individuals aged 18-45 years from Amazon Mechanical Turk (MTurk), an internet-based crowdsourcing platform that has been used successfully in a number of previous studies related to tobacco use and perceptions [6-10]. We targeted this age range to increase the probability of capturing individuals who use a variety of tobacco and nicotine products, given that prevalence tends to be higher among younger adults, particularly for emerging products [4,11]. Recruitment, informed consent, data collection, and remuneration take place entirely through the internet following the same procedures. We will describe the general protocol and address key differences across waves as necessary.

### Questionnaire

The Rutgers Omnibus consists of a core set of items that appear in all waves as well as rotating and 1-time supplemental question modules. The core items that appear in every iteration of the study include batteries on every and current (daily, some days, rarely, and past 30 days) use of cigarettes, electronic nicotine delivery systems, cigars (including traditional and premium, cigarillo, and filtered), smokeless tobacco (ie, moist snuff, chew, and snus), and modern nicotine products (eg, nicotine pouches). Each survey also contains items about product brands and flavors and exposure to tobacco advertising, an item capturing whether respondents have purchased tobacco or nicotine products that are different from their usual products in the past 30 days, and standard demographic questions. Rotating modules that are included periodically include question sets about cessation from tobacco use; product risk perceptions and social norms surrounding tobacco use; use of heated tobacco products, alcohol, and marijuana; use of coupons to purchase tobacco products; and mental health status.

One-time batteries may be added to a wave on an ad hoc basis for the purposes of generating pilot data or testing survey questions. Examples of such modules include split-sample experiments testing the impact of question wording, randomized experiments exploring variations in warning labels and ad features, and items that expand upon the core batteries by examining them in greater depth.

At all waves, the questionnaire is designed to take approximately 10 minutes to complete, depending on the number of products a respondent report using.

### Recruitment, Data Collection, and Data Quality

Omnibus participants are recruited from among eligible workers on MTurk using the MTurk Toolkit by CloudResearch (formerly known as TurkPrime), in order to enhance control over data quality [7]. CloudResearch regularly monitors MTurk worker engagement in order to identify individuals who are most likely to provide high-quality data. For all waves, we specify the following eligibility parameters: aged 18-45 years, located in the United States, and identified as high quality by CloudResearch-approved participant status. We further specify that CloudResearch should block workers coming from suspicious geocodes and duplicate IP addresses, as well as verify the country location of workers. Finally, CloudResearch ID numbers are assigned in place of participants’ Amazon ID numbers to ensure anonymous participation.

Individuals who meet all eligibility criteria receive an invitation in the form of an MTurk Human Intelligence Task. The Human Intelligence Task briefly describes our survey, and interested workers can choose to participate by clicking on a link to the survey, which is programmed in Qualtrics. Before beginning the survey, respondents must provide informed consent and verify their age. While the Omnibus is not designed to be a panel study, previous participants are eligible to complete a later wave.

Upon completing the survey, respondents are assigned a randomly generated redemption code, which they then submit to the CloudResearch platform in order to claim payment. Redemption codes entered on the platform are compared with the list of codes that were generated by Qualtrics to verify participation before payment is issued.

### Sample Size and Composition

At each wave, our primary goal is to collect high-quality data rapidly, to facilitate efficient monitoring of tobacco and nicotine product features and use. For this reason, we have no prespecified target sample sizes and instead target a fielding period of approximately 10 days. However, in waves that include a supplemental split-sample experiment, we have extended the fielding period in order to recruit enough participants to yield adequate statistical power, approximately 2500 participants. We decided to exit the field based on a combination of fielding duration, reaching a target sample size, and daily yield.

As the Omnibus is not primarily designed to be a panel study, a secondary goal at each wave is to maximize participation among eligible workers that have not taken part in previous waves of the study. CloudResearch enables the exclusion of individuals who have completed previous study waves. We have used this feature iteratively, whereby excluding some or all previous participants at a survey’s launch and slowly increasing the pool of previous participants, starting with the oldest iteration of the study (eg, allowing those who had participated in wave 1 but no subsequent waves), as the rate of daily participation declines.

### Ethical Considerations

The Omnibus protocol was reviewed by the Rutgers institutional review board and approved as exempt, category 2(i), because it involves ordinary survey procedures and data are collected such that participants’ identities cannot be readily ascertained. All participants are presented with an informed consent document, which includes that participation in the survey and responding to any questions therein are completely voluntary, before beginning the study; consent is indicated by clicking “I Agree” (clicking “I Do Not Agree” will terminate the survey). No identifying information is collected by the survey and CloudResearch ID numbers are assigned in place of participants’ Amazon ID numbers to ensure anonymous participation. Verified respondents in waves 1 through 6 were paid US $1.50 for completing the survey, which is consistent with similar MTurk tasks and a rate of approximately US $9 per hour; compensation was increased to US $2.00 beginning in wave 7 to combat slowed recruitment.

### Statistical Analysis

Although data collection is anonymous, we are able to identify unique (participated in 1 wave only) and repeated respondents (participated in multiple waves) according to participants’ anonymized CloudResearch ID numbers. Therefore, we are able to maintain single-wave, serial cross-sectional (all waves, unique individuals only), and longitudinal (all waves, linked by anonymous ID) datasets, enabling a variety of analyses including cross-sectional and longitudinal studies. At each wave, cost per complete was calculated by dividing the total amount paid to CloudResearch, including participant incentives and additional fees charged by CloudResearch, by the final number of participants for each wave of the survey.

## Results

The study launched in February 2022 and proceeded approximately quarterly thereafter. As shown in Table 1, the total number of participants ranged from 2082 (wave 8) to 2989 (wave 1), and the percentage of respondents who were new to the Omnibus survey, after wave 1, ranged from 70.5% (wave 2) to 19.9% (wave 6). The cost per complete at each wave was low, ranging from US $2.46 to US $3.27. The serial cross-sectional dataset for waves 1-8 contains 5822 participants, of whom all have participated in exactly 1 wave. The longitudinal dataset for waves 1-8 includes 10,334 participants, of whom 2477 had 3 or more data points.

Table 2 summarizes the demographic and tobacco use characteristics of Omnibus participants by wave, which have been largely consistent throughout the study. The majority of participants in each wave were aged 25-45 years, non-Hispanic White, and college educated, and about 55% to 60% were female. Roughly three-quarters of participants in each wave had ever smoked cigarettes, and prevalence of past–30-day smoking ranged from 32% to 36.3%. The trial of e-cigarettes was reported by nearly half of each wave’s participants (range 45.3%-49.9%), with past–30-day prevalence ranging from 19% to 24.4%. Of those who reported ever smoking cigars, almost 60% of each sample, past–30-day prevalence ranged from 12% to 14.8%. Smokeless tobacco product use was less common, with trial and past–30-day use ranging from 14.8% to 17.7% and from 3.3% to 5.6%, respectively. Finally, the nicotine pouch trial ranged from 5.5% to 8.5% over the 8 waves, while past–30-day use increased from 0.8% in wave 1 to 4.2% in wave 8.

**Table 1:** Fielding summary, Rutgers Omnibus Study waves 1-8 (February 2022 to December 2023).

Wave	Month and year	Total participants, N	New participants, n (%)	Cost per complete (US $)
1	February 2022	2989	2989 (100)	2.48
2	May 2022	2964	2091 (70.5)	2.49
3	August 2022	2526	1679 (66.5)	2.46
4	December 2022	2307	1243 (53.9)	2.46
5	February 2023	2530	877 (34.7)	2.46
6	May 2023	2518	501 (19.9)	2.46
7	August 2023	2174	443 (20.4)	2.87
8	December 2023	2082	503 (24.2)	3.27

**Table 2:** Sample demographic and tobacco or nicotine product use characteristics at each wave, Rutgers Omnibus Study waves 1-8 (February 2022 to December 2023)

		Wave 1 (n=2989), n (%)	Wave 2 (n=2964), n (%)	Wave 3 (n=2526), n (%)	Wave 4 (n=2307), n (%)	Wave 5 (n=2530), n (%)	Wave 6 (n=2518), n (%)	Wave 7 (n=2174), n (%)	Wave 8 (n=2082), n (%)
**Age range (years)**									
	18-24	323 (10.8)	308 (10.4)	283 (11.2)	239 (10.4)	230 (9.1)	210 (8.3)	176 (8.1)	144 (6.9)
	25-34	1363 (45.6)	1360 (45.9)	1142 (45.2)	1023 (44.3)	1074 (42.5)	1118 (44.4)	940 (43.2)	870 (41.8)
	35-45	1303 (43.6)	1296 (43.7)	1101 (43.6)	1045 (45.3)	1226 (48.5)	1190 (47.3)	1058 (48.7)	1068 (51.3)
**Sex**									
	Male	1297 (43.4)	1325 (44.7)	979 (38.8)	927 (40.2)	1110 (43.9)	1030 (40.9)	886 (40.8)	807 (38.8)
	Female	1691 (56.6)	1639 (55.3)	1546 (61.2)	1377 (59.8)	1419 (56.1)	1487 (59.1)	1288 (59.2)	1275 (61.2)
**Race and ethnicity**									
	Non-Hispanic White	1527 (68.2)	2026 (68.4)	1722 (68.2)	1547 (67.1)	1699 (67.2)	1687 (67)	1444 (66.4)	1400 (67.4)
	Hispanic	206 (9.2)	281 (9.5)	278 (11.0)	243 (10.5)	283 (11.2)	261 (10.4)	236 (10.9)	205 (9.9)
	Non-Hispanic Black	231 (10.3)	310 (10.5)	259 (10.3)	236 (10.2)	256 (10.1)	272 (10.8)	227 (10.4)	228 (11.0)
	Non-Hispanic Asian	161 (7.2)	209 (7.1)	146 (5.8)	152 (6.6)	174 (6.9)	168 (6.7)	143 (6.6)	126 (6.1)
	Non-Hispanic other	24 (1.1)	30 (1.0)	25 (1.0)	31 (1.3)	22 (0.9)	19 (0.8)	19 (0.9)	19 (0.9)
	Non-Hispanic multiracial	90 (4.0)	107 (3.6)	94 (3.7)	98 (4.3)	96 (3.8)	111 (4.4)	105 (4.8)	100 (4.8)
**Education**									
	High school graduate or below	387 (13.0)	395 (13.3)	316 (12.5)	312 (13.5)	316 (12.5)	324 (12.9)	309 (13.1)	269 (12.9)
	Associate degree or some college	947 (31.7)	979 (33.0)	916 (36.3)	811 (35.2)	813 (32.1)	816 (32.4)	719 (33.3)	697 (33.5)
	Bachelor’s degree	1185 (39.7)	1170 (39.5)	918 (36.4)	842 (36.5)	985 (38.9)	977 (38.8)	832 (38.4)	801 (38.5)
	Master’s degree or above	469 (15.7)	420 (14.2)	375 (14.9)	341 (14.8)	415 (16.4)	401 (15.9)	314 (15.2)	315 (15.1)
**Cigarettes**									
	Ever	2271 (76.0)	2242 (75.6)	1873 (74.2)	1694 (73.4)	1905 (75.3)	1888 (75.0)	1634 (75.2)	1554 (74.6)
	Past 30 day	1086 (36.3)	992 (33.5)	834 (32.0)	787 (34.1)	903 (35.7)	898 (35.7)	765 (35.2)	691 (33.2)
**e-Cigarettes**									
	Ever	1453 (48.6)	1478 (49.9)	1280 (50.7)	1150 (49.9)	1205 (47.7)	1196 (47.5)	973 (44.8)	943 (45.3)
	Past 30 days	707 (23.7)	707 (23.9)	615 (24.4)	561 (24.3)	599 (23.7)	573 (22.8)	468 (21.5)	396 (19.0)
**Cigars**									
	Ever	1795 (60.1)	1782 (60.1)	1483 (58.7)	1380 (59.8)	1484 (58.7)	1493 (59.3)	1219 (56.1)	1194 (57.3)
	Past 30 days	418 (14.0)	426 (14.4)	355 (14.1)	328 (14.2)	332 (13.1)	372 (14.8)	263 (12.1)	250 (12.0)
**Smokeless tobacco**									
	Ever	493 (16.6)	522 (17.7)	442 (17.5)	429 (18.6)	444 (17.6)	371 (14.8)	323 (14.9)	332 (16.0)
	Past 30 days	98 (3.3)	100 (3.4)	81 (3.2)	100 (4.3)	142 (5.6)	104 (4.1)	74 (3.4)	69 (3.3)
**Nicotine pouches**									
	Ever	165 (5.5)	217 (7.3)	147 (5.8)	165 (7.2)	156 (6.2)	155 (6.2)	156 (7.2)	178 (8.5)
	Past 30 days	25 (0.8)	79 (2.7)	53 (2.1)	64 (2.8)	86 (3.4)	78 (3.1)	68 (3.1)	87 (4.2)

## Discussion

### Principal Findings

The Rutgers Omnibus is fielded quarterly, beginning in February 2022, among an internet-based sample of US adults aged 18-45 years recruited from vetted MTurk workers through CloudResearch. Although a convenience sample, MTurk samples have fairly good geographic distribution, and the distribution of key demographic and socioeconomic characteristics are comparable to the US general population [6-8]. For the purposes of researching tobacco and nicotine product use, our MTurk samples have been particularly useful, with trial and past–30-day use prevalence exceeding those in national surveillance studies. For example, e-cigarette trial was reported by about 24% of the 2022 BRFSS sample, and current use by only 5%, as compared with nearly half and one-fourth of our samples, respectively [12]. Indeed, while Omnibus samples are not representative of all US adults, they are ideal for reaching individuals who use or are exposed to various emerging tobacco products. Moreover, we are able to recruit 2000 to 3000 participants for each wave within 10 days of fielding at a low cost and process the data for analysis immediately, making Omnibus especially useful for rapid monitoring and timely research of emerging products.

A key feature of the Rutgers Omnibus is our ability to generate both cross-sectional and longitudinal datasets while still maintaining the anonymity of participants. Although new workers join each month, the pool of eligible workers that have never participated in a Rutgers Omnibus survey shrinks with each iteration of the study. While including an increasing number of repeating participants requires vigilance for evidence of worker fatigue and panel conditioning [13], it has the advantage of producing data that can be used for multiple types of analyses.

The frequency and rapidity of the Omnibus study also make this a useful source of pilot data for project planning and grant proposals, as well as survey methods experiments. Requests to add supplemental measures to a specific wave of data collection, granted on an ad hoc basis, have resulted in several scholarly works and support for numerous grant proposals since the study’s inception [9,10].

### Limitations

This protocol is not without limitations. First, as a convenience sample of MTurk workers with unknown selection probability, the results of Omnibus analyses cannot be considered representative of all US adults or of adults aged 18-45 years. However, it is worth noting that such inferences are not necessary in the context of rapid surveillance when the goal is to identify signals rather than estimate population prevalence. In addition, representative samples are not necessary for survey experiment studies, where the focus is on maximizing internal validity, or for generating pilot data.

A second limitation is the potential for panel conditioning, especially as the pool of unique MTurk workers becomes smaller over time. However, existing evidence indicates that panel conditioning is most likely when study waves take place 1 month apart or less, and the literature on panel conditioning across study waves that are less frequent, such as ours, is divided [14]. To combat this, we have begun applying strategies to reduce the impact of conditioning, including diversification of questions wave to wave and reduction in survey length, with a focus on fielding rapidity over target sample size. As well, we have the ability to exclude repeated participants for primary or sensitivity analyses.

### Conclusions

The Rutgers Omnibus Study is a quarterly rapid survey that can also be leveraged to conduct survey experiments, generate pilot data, and address both cross-sectional and longitudinal research questions. Future waves of data collection will continue to follow this protocol.

## Abbreviations

BRFSS: Behavioral Risk Factor Surveillance System
MTurk: Amazon Mechanical Turk
NYTS: National Youth Tobacco Survey
PATH: Assessment of Tobacco or Health Study
TUS: Tobacco Use Supplement to the Current Population Survey

## Data Availability

The datasets generated during and/or analyzed during this study are not publicly available because our informed consent did not specify the secondary use of the data.
